# Supplementary material for: Splice-Junction-Based Mapping of Alternative Isoforms in the Human Proteome
Source: Cell Rep. Author manuscript; Available in PMC 2020 Jan 15. (PMC6961840; doi:10.1016/j.celrep.2019.11.026)
Supplement: 3 [file NIHMS1546469-supplement-3.zip › DF2/PXD009737/TestisTrypsin-525-Q9NYI0-AIMLLNTDLHGHNIGK.pdf]

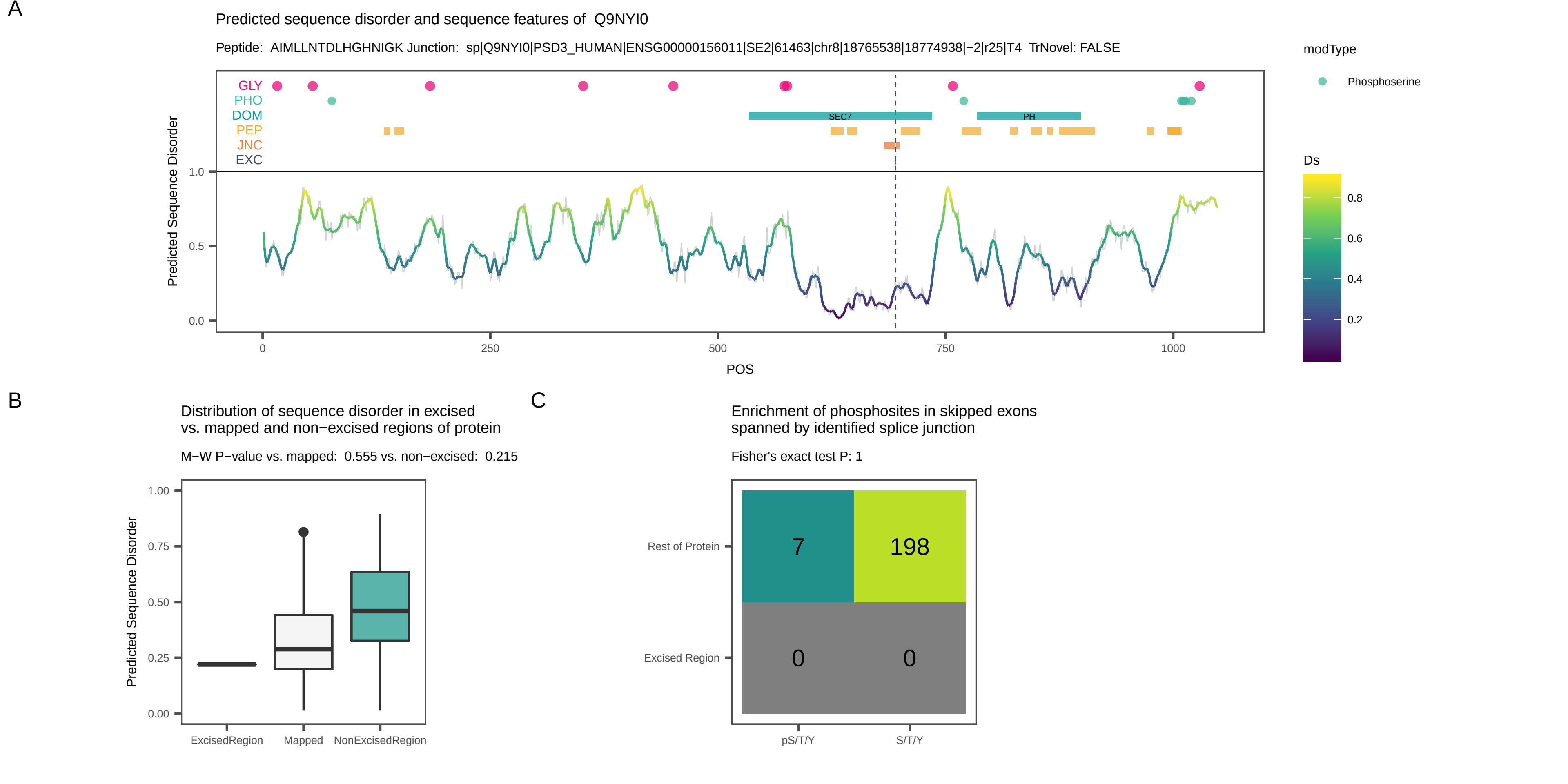
A
Predicted sequence disorder and sequence features of Q9NYI0
Peptide: AIMLLNTDLHGHNIGK Junction: sp|Q9NYI0|PSD3_HUMAN|ENSG00000156011|SE2|61463|chr8|18765538|18774938|−2|r25|T4 TrNovel: FALSE
GLY
PHO
DOM
PEP
JNC
EXC
SEC7
PH
Predicted Sequence Disorder
1.0
0.5
0.0
0
250
500
750
1000
POS
modType
Phosphoserine
Ds
0.8
0.6
0.4
0.2
B
Distribution of sequence disorder in excised vs. mapped and non−excised regions of protein
M−W P−value vs. mapped: 0.555 vs. non−excised: 0.215
Predicted Sequence Disorder
1.00
0.75
0.50
0.25
0.00
ExcisedRegion
Mapped
NonExcisedRegion
C
Enrichment of phosphosites in skipped exons spanned by identified splice junction
Fisher's exact test P: 1
Rest of Protein
Excised Region
7
198
0
0
pS/T/Y
S/T/Y
